# Gas Plasma Protein Oxidation Increases Immunogenicity and Human Antigen-Presenting Cell Maturation and Activation

**DOI:** 10.3390/vaccines10111814

**Published:** 2022-10-28

**Authors:** Ramona Clemen, Kevin Arlt, Thomas von Woedtke, Sander Bekeschus

**Affiliations:** ZIK plasmatis, Leibniz Institute for Plasma Science and Technology (INP), Felix-Hausdorff-Str. 2, 17489 Greifswald, Germany

**Keywords:** APCs, antigen uptake, dendritic cells, kINPen, monocytes, plasma medicine, reactive oxygen species, ROS

## Abstract

Protein vaccines rely on eliciting immune responses. Inflammation is a prerequisite for immune responses to control infection and cancer but is also associated with disease onset. Reactive oxygen species (ROSs) are central during inflammation and are capable of inducing non-enzymatic oxidative protein modifications (oxMods) associated with chronic disease, which alter the functionality or immunogenicity of proteins that are relevant in cancer immunotherapy. Specifically, antigen-presenting cells (APCs) take up and degrade extracellular native and oxidized proteins to induce adaptive immune responses. However, it is less clear how oxMods alter the protein’s immunogenicity, especially in inflammation-related short-lived reactive species. Gas plasma technology simultaneously generates a multitude of ROSs to modify protein antigens in a targeted and controlled manner to study the immunogenicity of oxMods. As model proteins relevant to chronic inflammation and cancer, we used gas plasma-treated insulin and CXCL8. We added those native or oxidized proteins to human THP-1 monocytes or primary monocyte-derived cells (moDCs). Both oxidized proteins caused concentration-independent maturation phenotype alterations in moDCs and THP-1 cells concerning surface marker expression and chemokine and cytokine secretion profiles. Interestingly, concentration-matched H_2_O_2_-treated proteins did not recapitulate the effects of gas plasma, suggesting sufficiently short diffusion distances for the short-lived reactive species to modify proteins. Our data provide evidence of dendric cell maturation and activation upon exposure to gas plasma- but not H_2_O_2_-modified model proteins. The biological consequences of these findings need to be elucidated in future inflammation and cancer disease models.

## 1. Introduction

The innate immune system is the first line of defense to protect the body from pathogens and their damage. During the local inflammatory reaction of an innate immune response, antigen-presenting cells (APC), such as dendritic cells (DC), randomly engulf proteins and present peptides to adaptive immune cells, such as lymphocytes. At the same time, the APCs become stimulated and activated, leading to a change in the surface marker expression and increased cytokine secretion. Another innate immune cell type entering the tissue during the first line of defense against invading pathogens is neutrophils. Neutrophils generate reactive oxygen species (ROS) and the heme enzyme myeloperoxidase (MPO), which in the presence of hydrogen peroxide (H_2_O_2_) generates the powerful oxidant hypochlorous acid (HOCl) and chloride ions (Cl^−^) [1,2,3,4]. H_2_O_2_ acts as a signaling molecule and chemoattractant to recruit immune cells [5,6]. Simultaneously, ROSs modify biomolecules, forming, e.g., dityrosine-containing cross-linked proteins, so-called advanced oxidation protein products (AOPP) [7]. Oxidative protein modifications in the microenvironment may simulate pro-inflammatory responses [8], and evidence has shown that ROS-treated proteins have altered immunogenicity. For instance, HOCl-treated protein ovalbumin (Ova) increases APC activation [9,10] and promotes APC-dependent activation of lymphocytes from transgenic animals [11]. In human type II-IV atherosclerotic lesions, oxidation and chlorination of laminin by MPO-derived ROS have been found [12]. However, short-lived reactive species, such as nitric oxide, peroxynitrite, and singlet oxygen, generate different oxidative modifications (oxMods) in proteins and lipids compared to long-lived species, such as H_2_O_2_ [13,14]. Hence, it is important to recapitulate the complex short- and long-lived ROS chemistry apparent during inflammation to infer about the biological consequences. Unfortunately, traditional chemical approaches allow generating only a limited number of types of ROS simultaneously.

Gas plasma technology generates various short-lived and long-lived ROSs simultaneously and without thermal harm. Gas plasma has various applications in physics, biomedical research, and medicine [15,16,17]. For instance, gas plasma was shown to have immunostimulatory properties. Gas plasma-treated APCs showed increased activation, characterized by migration and TNFα secretion [18], and upregulation of major histocompatibility complex (MHC-II) and other markers, such as CD83, CD86, CD40, CD25 [19,20], and CD63 [21]. Furthermore, successful immunization with gas plasma-treated tumor lysates exhibited increased immunogenicity compared to controls by protecting animals from tumor growth [22,23,24,25]. However, it is understudied if and to what extent gas plasma treatment affects protein immunogenicity and its inflammatory consequences in human APCs.

To this end, we investigated a human monocytic cell line (THP-1) and monocyte-derived APC (moDCs) regarding surface marker expression and secretion profile following incubation with the native, gas plasma-oxidized, and H_2_O_2_-oxidized model proteins CXCL8 (IL8) and insulin across different protein concentrations and using the well-characterized argon plasma jet kINPen [26]. H_2_O_2_ was used at the same concentration as the gas plasma treatment process. The equimolar H_2_O_2_ concentrations helped to understand whether the effects observed are unique to the gas plasma processes or can be mimicked using cheaper and more convenient chemical long-lived oxidants. Our results suggested that there are concentration-independent stimulatory effects of gas plasma-treated proteins, especially in moDCs, which could not be replicated by H_2_O_2_ treatment of the proteins.

## 2. Experimental Section

### 2.1. Monocyte Isolation and Cell Culture

Peripheral blood mononuclear cells were isolated from buffy coats dedicated to research and obtained at the Institute of Transfusion Medicine (Greifswald University Medical Center, Greifswald, Germany) via the Ficoll–Paque density gradient centrifugation method as described before [27]. Erythrocytes were lysed (RBC lysis buffer; BioLegend, Amsterdam, The Netherlands), and CD14^+^ monocytes were separated via positive magnetic bead separation (BioLegend). Cell purity was assessed using flow cytometry, and viable cell count was determined. A total of 2 × 10^5^ cells per well were seeded in a 24-well plate in 500 µL Roswell Park Memorial Institute medium (RPMI 1640; Corning, Kaiserslautern, Germany) containing 10% fetal bovine serum (FBS; Sigma-Aldrich, Taufkirchen, Germany), 1% L-glutamine (Corning), 1% penicillin/streptomycin (P/S; Corning), 800 international units human granulocyte-macrophage stimulating factor (GM-CSF; PeproTech, Hamburg, Germany) and 500 international units human interleukin 4 (IL4; PeproTech) to retrieve immature human monocytes-derived dendritic cells. Cells were incubated at 37 °C, 95% humidity, and 5% CO_2_. Human monocytic THP-1 cells (ATCC: Tib-202) were cultured in RPMI containing 10% FBS, 1% P/S, and 1% L-glutamine.

### 2.2. Proteins and ROS Treatment

CXC-motive-chemokine 8 (CXCL8), also referred to as interleukin 8 (IL8; BioLegend), or insulin (Sigma-Aldrich) was diluted in PBS and treated with ROS before THP-1 cells or moDCs were cultured with the proteins at indicated concentrations. PBS without proteins served as vehicle control. A total of 100 ng/mL, 1 µg/mL, and 10 µg/mL of IL8 or insulin in a total volume of 200 µL PBS were exposed to gas plasma for 60 s in a conductive mode (distance between nozzle and liquid surface: 5 mm). For gas plasma treatment, the atmospheric pressure plasma jet kINPen (neoplas, Greifswald, Germany) [26] was used with argon (purity 99.9999%; Air Liquide, Bremen, Germany) as the carrier gas. Gas flux-mediated evaporation of liquid was compensated for after the gas plasma treatment by adding a predetermined amount of double-distilled water to restore iso-osmolarity.

### 2.3. H_2_O_2_ Quantification

To generate so-called concentration-matched-controls (cmc), i.e., liquids containing the same concentration of H_2_O_2_ compared to a gas plasma-treated liquid in the absence of proteins, the concentration of H_2_O_2_ in gas plasma-treated PBS without protein was measured using the Amplex Ultra Red reagent kit (Thermo Fisher Scientific, Bremen, Germany) as described before [28]. For subsequent control experiments, chemically produced H_2_O_2_ (Sigma-Aldrich, Germany) was added to PBS freshly containing either IL8 or insulin on the day of experimentation to generate the cmc.

### 2.4. Flow Cytometry

Cells were collected in FACS tubes and washed three times with cold FACS washing buffer (Miltenyi Biotec, Teterow, Germany). Surface marker expression was investigated by incubating the cells with fluorochrome-conjugated antibodies (Table 1) after incubation with anti-CD16/32 Fc-block (BioLegend) for 10 min. Cell viability was determined using 1µM 4′,6-diamidino-2-phenylindole dihydrochloride (DAPI; BioLegend) or iFluor860 (Thermo Fisher Scientific). Flow cytometry experiments were performed using a CytoFLEX LX device (Beckman-Coulter, Krefeld, Germany), and data were analyzed using Kaluza software 2.1.3 (Beckman-Coulter).

### 2.5. Chemokine and Cytokine Measurements

Supernatants of stimulated cells were collected after 24 h incubation with proteins and stored at −20 °C until longitudinal analysis. Chemokines and cytokines were measured using multiplex cytokine detection technology according to the manufacturer’s instructions (BioLegend) and as described before [29]. Parallel quantification of a set of cytokines and chemokines (IFNα, IFNγ, IL1β, IL6, IL10, IL12p10, IL17A, IL18, IL23, IL33, MCP, and TNFα) was carried out using flow cytometry (CytoFLEX S; Beckman-Coulter). Data analysis was performed using LEGENDplex online software yielding absolute analyte quantities after normalization against a 5-log fitting of a standard.

### 2.6. Statistical Analysis

Graphing and statistical analysis were performed using *prism* 9.4.1 (GraphPad Software, San Diego, CA, USA). Comparison was made with different types of tests, as stated in the figure legends. Levels of significance (*p*-values) were indicated as follows: α = 0.05 (*), α = 0.01 (**), and α = 0.001 (***).

## 3. Results

### 3.1. IL8, but Not Insulin, Triggered Pro-Inflammatory Cytokines Secretion in THP-1 Cells

**Figure 1:**
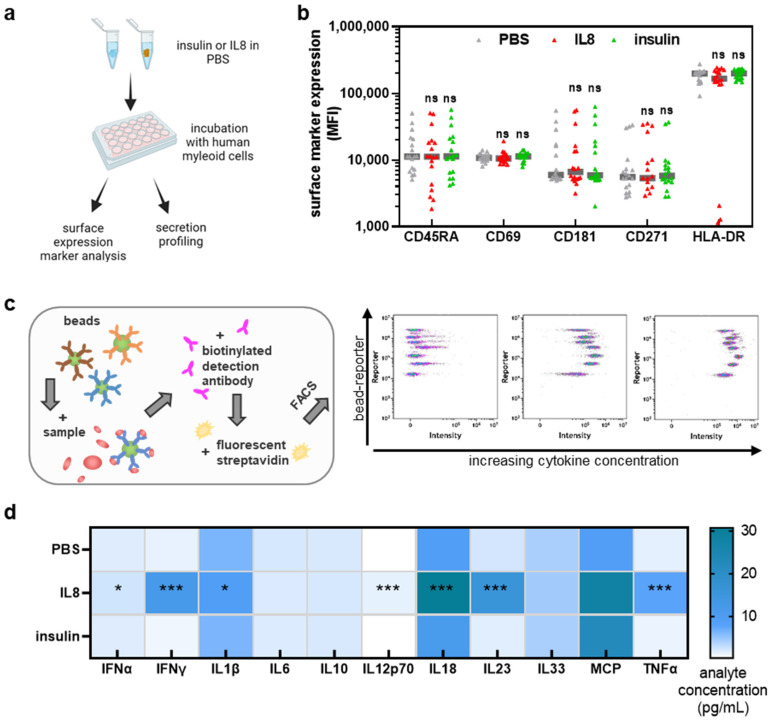
**IL8 but not insulin triggers the secretion of pro-inflammatory cytokines in THP-1.** (**a**) Workflow of the experimental procedure; (**b**) surface marker expression on IL8 or insulin-spiked THP-1 cells reveals no differences compared to unstimulated cells; (**c**) principle of bead-based multiplex assay; (**d**) absolute chemokine and cytokine levels of THP-1 cells exposed to IL8 or insulin. Data are the mean of at least three independent experiments. Statistical analysis was performed using two-way analysis of variances; for ANOVA: F = 2.8 (**b**)/33.2 (**d**), df = 2 (**b**)/2 (**d**). (* = *p* < 0.05; *** *p* < 0.001).

In this study, we aimed to understand the effect of the oxidation of two physiological model proteins, IL8 and insulin, in human APCs. Accordingly, it was assessed first if the native proteins confer effects in the cells (Figure 1a). THP-1 cells were harvested and stained to determine surface marker expression by flow cytometry analysis. No significant changes in activation markers HLA-DR, CD181, and CD69 were observed after incubation with native IL8 or insulin (Figure 1b). There was also no effect on the expression of the differentiation markers CD271 and CD45RA. We further investigated the supernatants of THP-1 cells by using bead-based multiplex cytokine analysis to quantify several analytes in parallel (Figure 1c). The addition of native (untreated) IL8 to the cell culture medium led to significantly increased release levels of several cytokines, namely IFNα, IFNγ, IL1β, IL12p70, IL8, IL23, and TNFα (Figure 1d). By contrast, the addition of surplus insulin to THP-1 cell cultures did not significantly affect the secretion of any of the analytes investigated when compared to PBS vehicle alone.

### 3.2. THP-1 Cell Incubation with Gas Plasma- but Not H_2_O_2_-Oxidized Proteins Increased Activation-Related Surface Marker Expression

**Figure 2:**
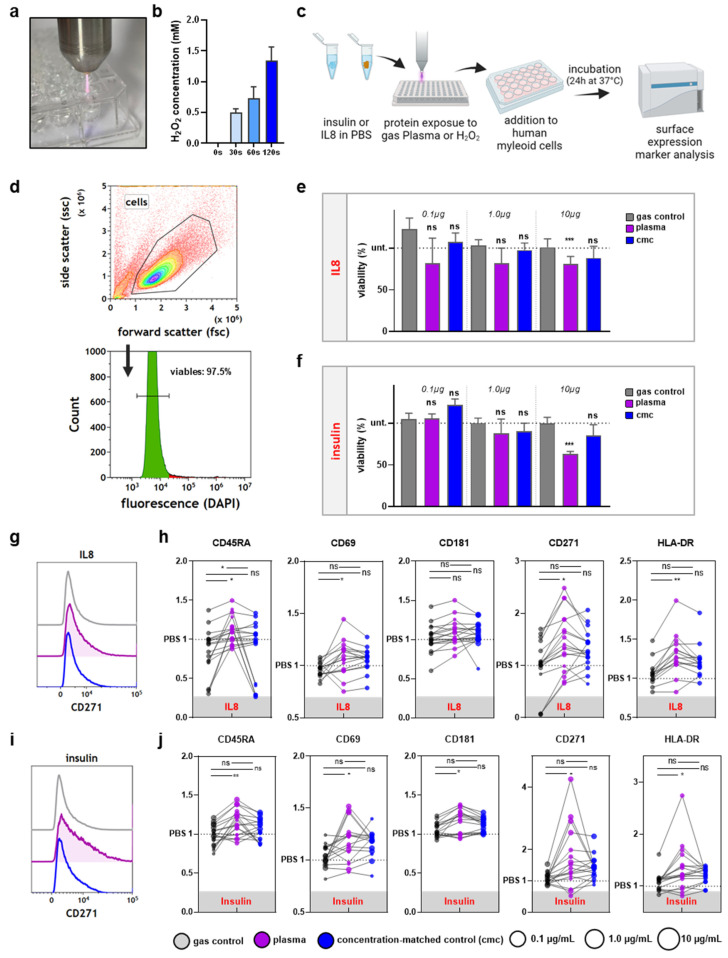
**Gas plasma but not cmc-treated IL8 and insulin stimulates THP-1 cells.** (**a**) Representative image of gas plasma treatment; (**b**) H_2_O_2_ concentration measured after exposure to gas plasma—the 30 s concentration was used as the cmc in subsequent experiments; (**c**) workflow for THP-1 incubation with native or oxidized proteins; (**d**) representative strategy of flow cytometry analysis; (**e**,**f**) quantified and normalized data of cell viability after incubation with native or oxidized IL8 (**e**) or insulin (**f**) concentrations; (**g**,**h**) representative histogram (**g**) and quantification of surface marker expression after incubation with native or oxidized IL8 (**h**); (**i**,**j**) representative histogram (**i**) and quantification of surface marker expression after incubation with native or oxidized IL8 (**j**). Data show individual technical replicates of an assay with three independent experiments. Data are the mean of at least three independent experiments. Statistical analysis was performed using one-way analysis of variances of gas plasma and H_2_O_2_ conditions against PBS (rhombi) or Mann–Whitney tests to compare gas plasma vs. H_2_O_2_ (asterisks); for ANOVA: F = 1.9 (0.1)/4.4 (1.0)/10.4 (10) and df = 2 (**e**); F = 3.4 (0.1)/2.4 (1.0)/23.0 (10) and df = 2 (**f**); F = 0.7 (CD181)/4.9 (HLA-DR)/4.2 (CD271)/4.4 (CD45RA)/4.0 (CD69) and df = 2 (**h**); F = 3.2 (CD181)/3.2 (HLA-DR)/4.2 (CD271)/6.1 (CD45RA)/4.1 (CD69) and df = 2 (**j**).

**Figure 3:**
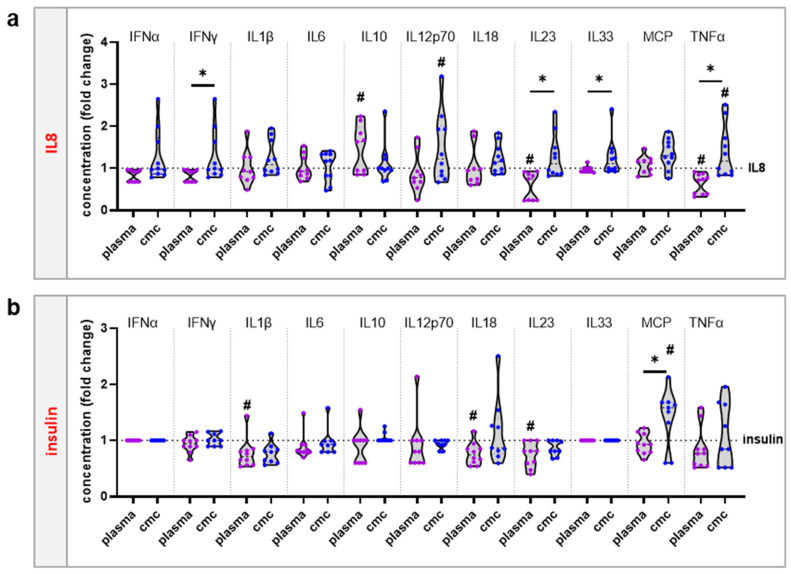
**Oxidized IL8 and insulin led to modestly altered secretion profiles in THP-1 cells.** (**a**,**b**) Secretion profiling was performed using bead-based multiplex assays of supernatants of THP-1 cells incubated with native or oxidized IL8 (**a**) or insulin (**b**). Data are the mean of at least three independent experiments. Statistical analysis was performed using one-way analysis of variances; for ANOVA: F = 3.0 (**a**)/2.6 (**b**), df = 2 (**a**)/2 (**b**). * denotes statistically significant differences between plasma and cmc (*p* < 0.05; *t*-test); ^#^ denotes statistically significant differences between the individual group and the gas insulin control normalized to 1 (*p* < 0.05; one-way analysis of variances).

**Figure A1:**
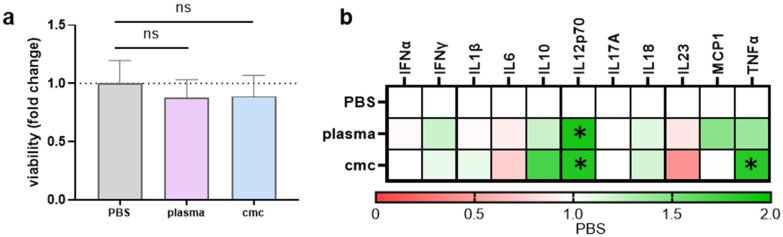
(**a**) Viability of THP-1 cells after incubation with protein-free PBS or gas plasma-oxidized protein-free PBS; (**b**) cytokine secretion of protein-free oxPBS-treated THP-1 cells normalized to secretion following protein-free PBS. Data are the mean of at least three independent experiments. Statistical analysis was performed using two-way analysis of variances; for ANOVA: F = 2.6 (**a**)/3.5 (**b**), df = 2 (**a**)/2 (**b**). ns = not significant; * denotes significant differences (*p* < 0.05).

To investigate the effect of oxidative protein modifications in THP-1, we next incubated the cells with insulin and IL8 after the treatment with gas plasma or H_2_O_2_. H_2_O_2_ is one of the most abundant ROS produced in gas plasma-treated liquids (Figure 2a). To generate an H_2_O_2_ control condition, the concentration of H_2_O_2_ in gas plasma-treated PBS was determined (Figure 2b). In subsequent experiments, a concentration-matched control (cmc) was freshly prepared, containing equimolar amounts of H_2_O_2_ in PBS compared to what gas plasma would generate in such liquid in the absence of proteins. The main difference between both treatments was that gas plasma also produced a range of short-lived reactive species. In the following experiments, cells were incubated with PBS containing IL8 or insulin and either exposed to gas plasma or supplemented with H_2_O_2_, and functionally tested (Figure 2c). After THP-1 cells with different concentrations were incubated for 24 h, cell viability was tested first (Figure 2d). Interestingly, gas plasma- but not H_2_O_2_-treated IL8 (Figure 2e) and insulin (Figure 2f) at the highest (10 µg/mL) but not medium (1 µg/mL) or low (0.1 µg/mL) concentrations conferred modest but significant cytotoxic effects in THP-1 cells 24 h after exposure. This, in principle, already indicated that the gas plasma treatment altered the proteins differently than the H_2_O_2_ exposure. In addition, the cytotoxic effects can be attributed to the proteins since neither gas plasma-treated PBS nor H_2_O_2_ on their own affected the viability of THP-1 cells (Figure A1a). Subsequently, the surface expression profiles were investigated in THP-1 cells exposed to IL8 (Figure 2g). In response to gas plasma-treated IL8, four out of five surface markers were significantly increased. In comparison, there was no significant increase or decrease following incubation with H_2_O_2_-treated IL8 (Figure 2h). Gas plasma-treated insulin was tested in the following (Figure 2i). All tested surface markers were significantly increased following incubation with gas plasma-treated insulin. In contrast, H_2_O_2_-treated insulin did not significantly affect the expression of any surface markers (Figure 2j). Surface marker expression changes often come with alterations in the chemokine and cytokine secretion profiles. Hence, we determined the effect of IL8 (Figure 3a) and insulin (Figure 3b) protein oxidation on the expression levels of 11 different analytes. Patterns between IL8 and insulin conditions for each of the specific analytes did not match; both proteins seemed to elucidate individual cellular responses. Gas plasma-treated IL8 significantly increased IL10 and decreased IL23 and TNFα release, while H_2_O_2_-treated IL8 increased extracellular IL12p70 and TNFα levels. Hence, gas plasma treatment of IL8 was suggested to have anti-inflammatory properties. The same profile, although for different analytes, was observed for gas plasma-treated insulin, significantly decreasing IL1β, IL18, and IL23 release levels. For H_2_O_2_, only an MCP1 increase was found. Of those data, only the changes observed with IL12p70 could be potentially attributed to the ROS produced due to the gas plasma treatment or the H_2_O_2_ itself (Figure A1b).

### 3.3. moDCs Activation and Secretion Profiles Were Profoundly Affected by Gas Plasma-Mediated Protein Oxidation

**Figure 4:**
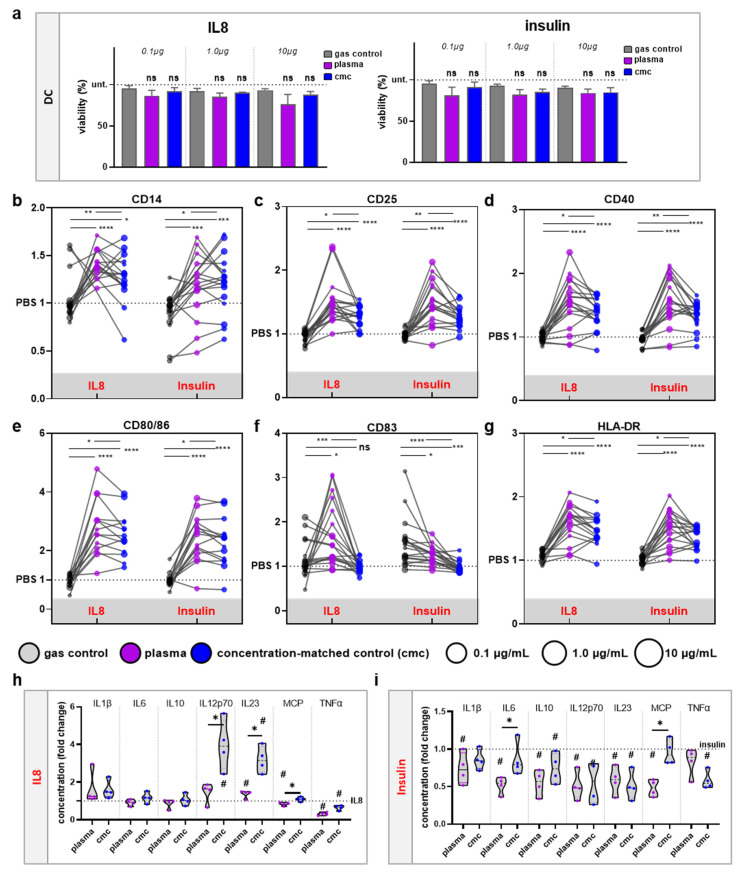
**Oxidized IL8 and insulin stimulate moDC activation and suppress cytokine secretion.** (**a**) Viability of human moDCs in response to native, gas plasma-, or H_2_O_2_-treated IL8 or insulin; (**b**–**g**) surface marker expression of CD14 (**b**), CD25 (**c**), CD40 (**d**), CD80/86 (**e**), CD83 (**f**), and HLA-DR (**g**) in moDCs incubated with the proteins was investigated using flow cytometry; (**h**,**i**) secretion profiling was performed using bead-based multiplex assays of supernatants of moDCs incubated with native or oxidized IL8 (**h**) or insulin (**i**). Data are the mean of at least three independent experiments. Statistical analysis was performed using one-way analysis of variances of gas plasma and H_2_O_2_ conditions against PBS (**a**–**g**: asterisks; **h**–**i**: rhombi) or Mann–Whitney tests to compare gas plasma vs. H_2_O_2_ (asterisks); for ANOVA: F = 2.8 (0.1)/1.5 (1.0)/3.9 (10) and df = 2 (**a**); F = 3.7 (0.1)/1.5 (1.0)/2.1 (10) and df = 2 (**b**); F = 27.4 (CD14)/64.2 (CD25)/68.6 (CD40)/88.5 (CD80/86)/13.6 (CD83)/87.0 (HLA-DR) and df = 2 (**b**–**g**); F = 2.5 (**h**)/19.7 (**i**), df = 2 (**h**)/2 (**i**). For (**b**–**g**): */**/***/**** denotes statistically significant differences between plasma and cmc, plasma and gas control, and cmc and gas control (* = *p* < 0.05; ** *p* < 0.01; *** *p* < 0.001; **** *p* < 0.0001); ^#^ denotes statistically significant differences between the individual group and the gas insulin control normalized to 1 (*p* < 0.05; one-way analysis of variances). For (**h**,**i**): * denotes statistically significant differences between plasma and cmc (*p* < 0.05; *t*-test).

THP-1 cells are a culture model for monocytes but lack certain features of primary cells [30]. At the site of inflammation, mononuclear phagocytes, such as immature dendritic cells and macrophages that derive from monocytes (moDC and moMac), take up proteins and differentiate after activation to promote T cell response. We isolated CD14^+^ monocytes and cultured them in GM-CSF and IL4 before adding native or gas plasma- or H_2_O_2_-oxidized IL8 or insulin for 24 h. The viability of the cells was not markedly affected by native or oxidized proteins, regardless of the concentration investigated (Figure 4a). With regard to surface marker profiling, gas plasma-treated IL8 and insulin significantly increased the expression of activation or maturation marker CD14 (Figure 4b), CD25 (Figure 4c), CD40 (Figure 4d), CD80/86 (Figure 4e), CD83 (Figure 4f) and HLA-DR (Figure 4g). Interestingly, in principle, the same results were retrieved for H_2_O_2_-treated IL8 and insulin. The difference was, however, that the responses observed for gas plasma treatment were significantly higher than when using H_2_O_2_ to oxidize proteins. This effect was true for all surface markers regardless of IL8 or insulin being investigated. These results suggest that moDCs respond sensitively to protein oxidation. As myeloid cell responses often come with alterations in the secretion profiles, several chemokines and cytokines were quantitatively assessed for IL8 (Figure 4h) and insulin (Figure 4i) culture conditions. Gas plasma-treated IL8 reduced secretion of MCP1 and TNFα and increased IL23 compared to native IL8. Interestingly, moDCs incubated with cmc-treated IL8 showed significantly higher levels of IL12p70 and IL23 than gas plasma-treated IL8. Strikingly, gas plasma-treated insulin showed remarkable effects in human moDCs, as all chemokines and cytokines (except for TNFα) investigated were significantly decreased. This was partially recapitulated for H_2_O_2_-treated insulin, which significantly differed from responses observed with gas plasma-treated insulin for a few of the analytes investigated. In summary, these data suggest that the incubation of a monocytic cell line or primary human monocyte-derived dendritic cells with gas plasma-treated IL8 or insulin promoted significant surface marker expression changes along with altered patterns of chemokine and cytokine release, which may be attributed to the perception of oxidized proteins as a danger signal, with important implications for testing protein immunogenicity in terms of vaccination campaigns.

## 4. Discussion

There is significant interest in understanding the functionality and immunogenicity of ROS-modified structures and the association between such oxMods and pathology [31,32,33,34,35]. At the same time, understanding the immunogenicity of protein oxidation has important implications for vaccination. We here use gas plasma technology for generating highly reactive radicals and short-lived reactive species to investigate the effect of oxidized proteins on antigen-presenting cells.

During an inflammatory response, neutrophils generate the oxidizing hypohalous acids HOCl, HOBr, and HOSCN by the enzyme myeloperoxidase [3]. Furthermore, enzymes such as NADPH oxidases, xanthine oxidase, and 5-lipoxygenase can rapidly increase local H_2_O_2_ levels [36]. The major source of ROS in the TME is myeloid cells, such as macrophages and neutrophils [37]. They cause their effect through the so-called oxidative burst the biological system in general and local proteins in particular. Consequently, oxidative post-translational modification of proteins can lead to altered activity, functionality, and immunogenicity. For instance, the biological activity of HMGB1 depends on its redox state being either oxidized or reduced, ranging from pro- to anti-inflammatory [38]. The altered immunogenicity caused by oxidized amino acids or structural changes plays an important role in diseases by affecting immune cell activation. For instance, H_2_O_2_-oxidized β-glycoprotein I (β_2_-GPI) and naturally oxidized β_2_-GPI were shown to increase the expression of the DC activation marker HLA-DR, CD40, CD80, CD83, and CD86 [39]. In line with those results, we also observed increased surface marker expression when moDCs were incubated with oxidized proteins. A plausible mechanism involves the adjuvant effect of oxidized structures, leading to increased immunogenicity. Indeed, HOCl-modified serum albumin inhibits the uptake of other antigens and IFNγ-mediated MHC-II expression in THP-1 derived M1 macrophages [40]; the authors suggest that N-chlorinated protein competes with the other antigen for the binding to the same receptor. Biedron and colleagues observed reduced antigen uptake of bone-marrow-derived dendritic cells when the cells were previously incubated with HOCl-modified proteins [9]. We reported similar findings on the preferred uptake of gas plasma-treated proteins in a competition assay, where human moDCs were incubated with oxidized proteins and fluorescent proteins [41]. It should be mentioned here, however, that argon gas plasma was used in the present study, in which no HOCl but primarily H_2_O_2_ was generated.

Interestingly, a generalized activation by oxidized proteins was not found in our work, as H_2_O_2_ treatment did not reproduce the effects of gas plasma-treated proteins entirely and throughout all investigations in our study. Along similar lines, IL8 and insulin did not produce the same effects. The first obvious reason for this is the different physiological functions of both proteins, likely contributing in their own way to the results observed. A second reason could be their different structures and varying amino acid compositions and sequences. Several receptors were proposed for recognizing oxidized proteins, namely toll-like-receptor (TLR), mannose receptor (CD206), scavenger receptors A (CD204/ SR-A), CD36, SREC-I, and lectin-like oxidized low-density lipoprotein receptor (LOX-1), as well as RAGE [9,10,35,39,40,42]. Here, however, it should be taken into account that the different receptors were examined in other phagocytic cells. Our study also observed other effects of oxidized IL8 and oxidized insulin on THP-1 and moDCs. This is not much of a surprise, starting with the fact that we left THP-1 undifferentiated, i.e., without incubation of cytokines prior to incubation with the proteins, while moDCs were incubated with IL4 and GM-CSF, apart from intrinsic differences observed with both cell types [30]. Our data also support previous reports, where differences in phenotypic changes, activation, and maturation in moDCs, THP-1, KG-1, and MUTZ-3 after incubation with proteins or chemical sensitizers were shown before [43,44,45,46]. However, different activation and maturation abilities of the different cell types also point to the presence of different subpopulations in the organism and during an immune response that need to be considered in general. This makes studying oxidized proteins in the inflammatory context more complex, and further comparative studies are needed.

In our study, we did compare not only THP-1 versus moDCs but also different proteins and different concentrations. We did not observe a correlation between protein concentration and APC activation in both cell types. However, a correlation between ROS concentration for oxMod-generation and immunogenicity was shown previously in vivo [11]. More research must be performed to investigate the effects of ROS concentrations and a variety of ROS on proteins produced by gas plasmas; parallel studies on the effect of gas plasma-induced oxMods on immune cells help to understand the individual recognition, signaling, and immune responses associated with them. Gas plasma is tunable, and different ROSs are generated, depending on different feeding gas and gas mixtures, which were previously shown to have different effects on the immunogenicity of Ova [41]. Furthermore, treatment conditions, such as exposure time or conductive mode, influence ROS generation and potential protein oxPTMs [47].

In summary, we observed different effects of protein oxidation and immunogenicity, depending on the type of protein, exposure to H_2_O_2_ or ROS cocktail, and cell type. Indeed, we determined different effects of oxidized IL8 and oxidized insulin on THP-1 and moDCs, although different protein concentrations did not influence activation and maturation. Furthermore, oxidation by gas plasma seemed superior to using H_2_O_2_ alone, while both had specific effects on the cells. Our results suggest that different oxidative post-translational modifications induced by various ROSs lead to distinct recognition, further processing, and the activation/ maturation state in APCs, which, ultimately, also depends to a certain extent on the protein investigated.

## 5. Conclusions

Oxidative stress and ROS generation during inflammation lead to advanced oxidation protein products and oxidized proteins. Phagocytic immune cells take up extracellular oxidized proteins, become activated, and maturate to support an adaptive immune response. Gas plasma technology generates a variety of ROSs simultaneously and is a promising tool for investigating protein oxidation and immunogenic effects. We studied the effect of gas plasma-treated IL8 and insulin on phenotypic alterations and cytokine secretion profiles in moDCs and THP-1 compared to H_2_O_2_-tested proteins. Taken together, the results show different immunogenicities of exogenous ROS-induced oxidized proteins. Furthermore, we observed significant differences in moDCs while THP-1 cells were more reserved in response to oxMods.

## Figures and Tables

**Table 1:** Fluorescently-labeled monoclonal antibodies used in this study.

Ligand	Fluorescent Label	Clone	Supplier
CD11C	PE-Cy7	S-HCL-3	BioLegend
CD14	BV650	M5E2	BioLegend
CD25	AF488	BC96	BioLegend
CD40	APC	HB14	BioLegend
CD45RA	PerCP/Cy5.5	HI100	BD Biosciences
CD69	AF488	FN50	BioLegend
CD80	PE	2D10	BioLegend
CD83	PerCP/Cy5.5	HB15	BioLegend
CD86	PE	BU63	BioLegend
CD181	PE	8F1/CXCR1	BioLegend
CD271	PE-Cy7	C40-1457	BD Biosciences
HLA-A,B,C	AF700	W6/32	BioLegend
HLA-DR	APC-Cy7	L246	BioLegend

## Data Availability

The underlying data from this work can be retrieved from the author upon reasonable request.
